# End-of-life care for people with advanced dementia and pain: a qualitative study in Swedish nursing homes

**DOI:** 10.1186/s12912-021-00566-7

**Published:** 2021-03-20

**Authors:** Emma Lundin, Tove E. Godskesen

**Affiliations:** 1 Nacka Senior Centre Talliden, Helgesons väg 5, SE-131 37 Nacka, Sweden; 2 Centre for Research Ethics & Bioethics, Uppsala University, BMC, Box 564, SE-751 22 Uppsala, Sweden; 3 Department of Health Care Sciences, Palliative Research Centre, Ersta Sköndal Bräcke University College, Box 11189, SE-100 61 Stockholm, Sweden

**Keywords:** Dementia, Pain, End-of-life, Caring, Nursing home, Nurses, Education

## Abstract

**Background:**

Of the Swedish people with advanced dementia, the majority die in nursing homes. Unresolved pain can occur in people with a terminal illness such as dementia. However, pain management in people with advanced dementia is often suboptimal and inadequate, with fewer palliative care interventions than offered to cancer patients. Although they are largely responsible for the care of these people, few studies have addressed the experiences of registered nurses in this respect. Therefore, the aim of this study was to describe the experiences of nurses in caring for people with advanced dementia and pain at the end of life.

**Methods:**

The study had a descriptive explorative design. Individual qualitative, semi-structured interviews were carried out with 13 nurses from 12 nursing homes in Sweden. The results were analysed using thematic content analysis.

**Results:**

The nurses described communicative, relational and organisational challenges. One major issue involved difficulties communicating with the person with advanced dementia, resulting in uncertain pain assessment. Other difficulties involved the differentiation of pain from anxiety, the balance of benefits and risks with morphine administration, and the creation of good relationships with healthcare personnel and the persons’ relatives. Relatives can greatly affect the assessment and management of pain, both because of their ability to interpret pain behaviour and by questioning the care given. Good pain management was facilitated by good communication and relationships with healthcare staff and relatives, extensive professional nursing experience, and already knowing the person with advanced dementia.

**Conclusions:**

This study highlights the need for nursing homes to employ specialist nurses who have been trained in the appropriate knowledge and skills to deal with the challenges of end-of-life care for people with advanced dementia and pain. Additionally, there should be resources and strategies available for providing information to family members and for involving them in the decision-making process, as they are often unfamiliar with the multitude of considerations involved in decisions such as whether to administer morphine or not.

**Supplementary Information:**

The online version contains supplementary material available at 10.1186/s12912-021-00566-7.

## Background

Dementia is a progressive, untreatable condition that includes cognitive impairment, neuropsychiatric symptoms and loss of independent function. Associated with it are complex needs with high levels of dependency and morbidity, especially in advanced dementia. There are about 50 million people with dementia worldwide and the number is expected to triple by 2050 [1]. In Sweden today, approximately 140,000 people are living with dementia [2]. The incidence rises exponentially for those aged between 65 and 90, doubling approximately every 5 years [3]. Sweden has one of Europe’s fastest-growing populations of elderly citizens, where one in five is 65 or older, therefore an increased dementia-based public health burden can be expected. People with dementia tend to want to stay at home, but as their condition progresses and they experience the need for full-time care, many moves into nursing homes. The majority of nursing homes in Sweden have beds both for people with physical diseases and for those with dementia, and more than half of the nursing homes have specialised dementia units [4]. Currently, almost half of the Swedish people with advanced dementia are being cared for in a nursing home [4]. The county councils are responsible for providing residents with medical care and the local municipalities for care of elderly in the home or at special accommodations, such as nursing homes [5]. Physicians are not usually employed by the nursing home but visit for a period each week. Due to physicians’ shortage, geriatricians from geriatric clinics often provide medical and geriatric care at nursing homes [6]. Therefore, registered nurses [termed nurses from here on] have to assume responsibility for the care of these persons, who often have complex health problems [7]. Nurses also play an active and important part in assessing and relieving pain and suffering [8]. At nursing homes, primary care physicians are in charge of residents’ care.

Palliative care for people at the end of life is in Sweden divided into general and specialised palliative care [9]. General palliative care is provided to patients whose needs may be met by staff with basic caring knowledge. Such care is conducted in municipal care and caring homes in collaboration with primary care. Specialised palliative care is provided to patients with complex symptoms or special needs, conducted by a multi-professional team with specialised knowledge and expertise in palliative care. This care is offered within a specialised palliative care unit or in advanced home care. Persons with advanced dementia at the end of life are given general palliative care at regular nursing homes.

People with advanced dementia in their final phase of life have complex needs and, as they gradually become more and more dependent, the need for nursing care increases [10]. Like the rest of the elderly population, people with dementia struggle with physical challenges such as rheumatoid arthritis, which causes considerable pain. When the person is nearing the end of their life also recurrent infections and/or pressure ulcers can sometimes be painful. Additionally, they have a higher burden of co-morbid physical disease and a greater symptom burden than people without dementia while also struggling to verbalise [11, 12]. For example, pain, shortness of breath and agitation are common symptoms or consequences of comorbidity that increase in people with dementia shortly before death [13]. If the person has other life-limiting conditions that can cause pain, such as cancer, their condition is likely to worsen more predictably [14–16].

Studies have shown that persons with dementia living in nursing homes risk receiving suboptimal pain relief [13, 17]. Nurses can assess pain through a variety of pain assessment tools [18], but the quality and utility of these tools have been questioned [19–21]. Further, opioids are usually the first choice for analgesia at the end of life [22], but there is evidence that those with advanced dementia respond poorly to opioid treatment and experience more harmful side effects than people without dementia [22, 23]. Since persons with dementia at the end of life find it difficult, or are unable, to self-report pain, many of the available pain assessment tools cannot be used [24]. Therefore, suitable pain assessment tools for this population have been developed, such as Abbey Pain Scale and Doloplus 2.

Nurses in nursing homes play a crucial role in pain management for residents with dementia at the end of life, but few studies have elicited their perspectives in this context. Therefore, the aim of this study was to describe the experiences of nurses caring for residents with advanced dementia and pain at the end of life.

## Methods

A descriptive explorative study design was used. Data were collected from qualitative semi-structured interviews. This data collection method allows participants to provide more information, such as feelings and attitudes, and their understanding of the subject [25]..

### Settings and participants

All participants were recruited from nursing homes units specialised in dementia care in Stockholm, Sweden. Inclusion criteria were at least 1 year of practice as a registered nurse in a nursing home and experience of end-of-life care for residents with advanced dementia and pain.

Sixteen of the forty-six administrative managers at the randomly chosen nursing homes permitted the study. These managers, on behalf of the researchers, distributed a letter of invitation and information about the study to nurses who met the inclusion criteria. The high workload, on-going reorganisation of the wards, and requirement for extensive experience of end-of-life care for residents with advanced dementia and pain made recruitment of a sufficient number of participants challenging. The sample consisted of 13 nurses from 12 nursing homes: ten women and three men (Table 1). The participants had an average of 10 years’ experience in dementia nursing care, ranging from one to 15 years. Eleven nurses had a bachelor’s degree in nursing, while two had graduated earlier when training comprised a two-year programme. None had specialist training in palliative, geriatric or dementia nursing.

**Table 1:** Characteristics of the participant nurses (*n* = 13)

**Gender**	
Female	10
Male	3
**Age (years)**	
Range	< 27 to > 66
< 29–39	2
40–49	1
50 to > 66	10
**Education**	
Bachelor’s degree (3-year)	11
Nursing programme (2-year)	2
Specialist nursing degree in palliative, geriatric or dementia nursing	0
**Years working as a nurse**	
Range	2 to 37
Mean/median	21/20
**Years working in dementia ward at a nursing home**	
Range	1 to 15
Mean/median	10/10

### Ethical considerations

Ethical approval for site participation was not obtained, as this is not required in Sweden for non-interventional research which poses no identified risks for study participants and does not require the processing of sensitive data [26]. Nevertheless, the study followed accepted ethical standards, as outlined in the Declaration of Helsinki [27]. As part of the consent process, participants were informed that participation was voluntary and that consent could be withdrawn at any time without explanation. Participants were also assured, in both written and oral form, that data would be handled confidentially and that the results would be reported in such a way that identification of the informants was not possible. The collected data were handled in line with the EU General Data Protection Directive. All identifiers were removed from the transcripts before they were distributed to the research group and the data were stored securely in a password-protected computer. Signed consent forms were collected before inclusion.

### Data collection

Individual participants were interviewed according to an interview guide (see Supplementary file 1). The guide was developed from a review of the literature and the questions were guided by the study aim. The questionnaire consisted of semi-structured, open-ended questions, with follow-up questions for further clarification. Before the interview, the interviewer informed the participants that the questions would be focused on the last weeks and days before the persons’ death. A pilot interview to test intelligibility and suitability led to minor adjustments. After oral and written consent, individual interviews by the first author were scheduled between February and April 2018. The digitally recorded interviews took place during work hours at the nursing homes. They lasted between 10 and 32 min, with an average of 21 min. No prior relationship existed between the nurses and researchers.

### Data analysis

The data were analysed using thematic content analysis [28]. To obtain an overall view of the data, one of the researchers (EL) read the verbatim transcriptions repeatedly. Short notes (codes) in the margins were used to summarise the content. Next, distinct categories were formed by searching for similarities and differences among the notes. Then the number of categories was reduced by merging similar ones and discarding those not relevant to the study. Lastly, the data collected for each category were colour-coded and copied into the relevant page. Patterns between the categorised pages led to the formulation of themes (Table 2).

**Table 2:** Example of the analysis process

Meaning units	Codes	Category	Theme
When they cannot express if they are in pain nor grade their pain, then it is about me and my ability to assess their expressions, to look at face expressions and body language, movements, pallor or cold sweat and physical signs...you need to understand if it is about pain or if it is about anxiety or if it is a combination.	Interpretation of pain	Uncertainties when assessing pain	Communication challenges: assessing and relieving pain
	Signs of pain		
	Lack of verbal communication		
	To distinguish pain from anxiety		

## Results

The results described the nurses’ experiences of end-of-life care for residents with advanced dementia and pain, using three themes: communication challenges, relational challenges, and organisational challenges.

### Communication challenges: assessing and relieving pain

#### Uncertainties when assessing pain

Many nurses found it challenging to assess pain. A common cause of uncertainty was when the cognitive impairment of dementia affected the person’s ability to verbally express if they experienced pain, which becomes a barrier to assess if pain medication has achieved an optimal level of pain relief. As one nurse said: “It is difficult, because most of them you cannot ask and get a clear answer from” (Nurse 11). The nurses thought that asking the residents with advanced dementia about their pain was the first step in pain assessment. When verbal communication was impossible, the nurses then focused on the interpretation of pain expressions: verbal expressions such as groaning and screams, and nonverbal expressions such as anxious behaviour and body language including facial expression. Several nurses found distinguishing pain from anxiety challenging: “It is really hard to assess pain and it is not easy to interpret whether it is about pain or anxiety” (Nurse 6). The similarity of expressions of pain and anxiety made the nurses feel uncertain.

Another communicational challenge that caused uncertainty was if the nurses lacked an earlier relationship with the residents with advanced dementia. That made pain assessment more difficult; for example, it is then impossible to compare current behaviour with past behaviour. As expressed by one nurse:

It felt tough because I didn’t know her from before...I couldn’t know if she had behaved like this before and that made it difficult. It helps so much if you have been taking care of the person for a while. (Nurse 10)

As lack of verbal communication was the primary reason for difficulties with pain assessment, several nurses emphasised the importance of communication skills and suggested that professional experience developed their abilities:

It demands skill to be able to assess whether a person with dementia is in pain ...you need to be really good at communication to sort this out...and this is something you have to learn through practice. (Nurse 5)

Most of the nurses used the Abbey Pain Scale assessment tool. However, some of the nurses did not use any assessment tools at all, believing that the available tools were inadequate or that there were no guidelines at all. Instead, these nurses assessed pain by intuition.

#### Uncertainties when administering pain relief

Similarly, these nurses used an intuitive approach to decide the morphine dosage. This situation made it hard to achieve the correct balance between optimal pain control and optimal safety.

Safety is an issue because the most commonly used pharmacological treatment is subcutaneous morphine by injection. The nurses found it difficult to achieve the right dosage balance, avoiding doses that were too small for adequate pain relief and those that were so generous that respiratory depression resulted. As one nurse said:

Then one is a bit careful as morphine is a problem, i.e. it affects the respiratory centre … One does not want to sedate someone, despite having reached the end of life – you try to give just the required pain relief... (Nurse 6)

Thus, the nurses tried to find the dose that made the person peaceful and pain-free and felt satisfaction when they succeeded. As one nurse described it: “It’s really difficult to find a balance, to find the exact dose ... it is very positive seeing a person relaxing and not expressing pain” (Nurse 7). Despite worries about respiratory depression, several nurses believed it was better to administer morphine generously and preferable to give a bit too much rather than too little: “Well, yes, my point of view is that it is important to give something, and rather a bit too much than too little” (Nurse 11).

The difficulty of distinguishing pain from expressions of anxiety made several nurses adopt the strategy of combining morphine injections with midazolam injections for anxiety. As one nurse expressed it:

I noticed I had to relieve both pain and anxiety and he was so tense before and after injections of morphine and midazolam he got so relaxed; they go hand in hand, pain and anxiety...it is not unusual that we combine medications for pain and anxiety. (Nurse 8)

### Relational challenges: the influence of relatives

Mostly, relatives were perceived as a positive factor when caring for the dying person with advanced dementia, but they could sometimes negatively influence the administration of analgesics. Several nurses viewed relatives as significant resources in pain assessment, as relatives were often familiar with the earlier behaviour of the residents with advanced dementia and could interpret their behaviour for the nurses. One nurse said that a grandchild had been “really alert when she sat beside her grandmother’s bed; she immediately signalled to us when she saw signs of pain” (Nurse 1). The presence of relatives beside these residents with advanced dementia is important in itself, and they were usually called in by the nurses when they were approaching death.

Family members sometimes asked for an ongoing relationship with the healthcare staff, so they could actively influence and help with the care. At other times relatives could pose a challenge; this could happen, for example, when the person was administered analgesics. The relatives could be uncertain about or even afraid of morphine, and “the atmosphere in the room can change immediately when morphine injections are about to be administered” (Nurse 10). If relatives believe that their next-of-kin is not adequately relieved of pain, nurses often comply with their wishes for more pain medication:

Relatives often want the person to receive a lot of analgesics; of course, they don’t want them to suffer, and sometimes you listen to them, and sometimes you give an injection maybe more for the sake of calming down the relatives a little, to satisfy them, to make them feel at ease. (Nurse 9)

In some cases, relatives reportedly insisted on sending the person to the hospital because they thought that they were not adequately relieved of their pain. In other cases, relatives at the bedside asked the nurse to give as little morphine as possible, either because they were afraid that the drug could provoke the person’s death or because they feared that the person could become addicted. Relatives’ attitudes toward morphine were often mentioned as the most complicated issue related to pain medication. This often caused stress or, as one nurse put it: “It’s a pressure, to be surrounded by questioning relatives” (Nurse 5). The nurses thought that the whole process of administering morphine could be strongly facilitated if they communicated closely with the relatives, exchanged information with them, and gave them good reasons for administering morphine.

### Organisational challenges

#### Time constraints

Several challenges at an organisational level were mentioned by nurses as barriers to the provision of good quality, person-centred palliative care for those with advanced dementia. Several nurses emphasised the importance of being readily available in order to relieve the pain effectively. This can be crucial, as having nurses present “can relieve pain that comes from anxiety and loneliness” (Nurse 6). If residents with advanced dementia are left alone, they can feel lonely, which can create or increase physical and psychosocial pain. As one nurse expressed it:

Many things are important when it comes to pain, not least the psychological part. If a person feels exposed and feels like no one is there and no one cares about them, then I think they will feel more pain. (Nurse 9)

Many nurses reported that understaffing, limited time and heavy workloads made it difficult to provide adequate care and attend properly to residents with advanced dementia. The nurses wanted healthcare personnel to stay with the dying person when relatives were not present. However, sometimes the workplace did not permit additional healthcare personnel because of economic barriers, causing the person with dementia to die alone and in pain. This made the nurses feel powerless and helpless: “It is miserable to know that a dying person is alone and has nobody present ... because staff presence is insufficient” (Nurse 6). Their heavy workloads often prevented nurses from staying with the person who was dying:

We nurses are so few that we don’t have the time to sit and hold someone’s hand and try to comfort them ... most of the time we are sitting down documenting [the work]. I hardly find time to be among the residents. (Nurse 7)

Time constraints were commonly described as a significant barrier to the nurses being readily available to persons with advanced dementia. This was commonly lamented: “If I could wish for something, it would be more staff and more resources” (Nurse 13).

#### Lack of knowledge and competence

Another organisational challenge often mentioned by nurses was the apparent lack of professional competence and ability to apply a palliative care approach according to the philosophy of palliative care. All healthcare personnel were seen as necessary by nurses but sometimes when healthcare personnel lacked sufficient competence (such as sensitive awareness or an appropriate personal attitude) to handle the complex palliative care demands, it could become challenging. As one nurse said:

I have seen that persons with dementia can be more or less calm according to which staff are working … If there are anxious staff working, so to speak, if they talk loud, if they are stressed or something, then the person with dementia can also become worried and experience more pain … Meeting people with dementia is an art that is so important – to calm them and treat them with respect. (Nurse 9)

The nursing homes in this study were staffed by physicians who did not spend the majority of their practice caring for persons with dementia. The physicians often stated that there was no maximum morphine dose but that it was important to start with a small dose and then titrate it until they were comfortable. Many nurses in this study were responsible for determining the amount and frequency of morphine administration, even though no nurses had specialist training:

… Most often doctors prescribe no upper limit for the total administered dose, which means that I as a nurse have the greatest responsibility here when it comes to pain relief at the end of life. (Nurse 13)

Some nurses felt that this was a challenge and that the responsibility for morphine became greater during palliative care, with increased demands on the nurse’s knowledge and competence.

Lastly, the nurses mentioned that they often co-operated with healthcare personnel and that they found this very important as those personnel often cared more directly for the person with dementia and therefore were better able to perceive their pain. On the other hand, healthcare personnel often lacked essential knowledge or experience relating to pain management:

“Lack of knowledge is one reason for it not working so well with healthcare personnel ... knowledge improves everything and provides the prerequisites.” (Nurse 12)

Some nurses experienced difficulties with stand-in or temporary healthcare personnel as they did not know the person with advanced dementia and were unaware of their baseline behaviour. This increased the risk of missing signs of pain and not reporting them to the nurses, as one nurse described:

“When I entered the room, I noticed the person was really in pain, and I questioned the healthcare personnel: why haven’t you said something to me?” (Nurse 9)

## Discussion

This study found that nurses caring for residents with advanced dementia and pain at the end of life are facing communication, relational and organisational challenges.

### Communication challenges

Although unresolved pain can occur in residents with advanced dementia at the end of life, pain relief is a priority in palliative care; however, nurses reported that, as persons with advanced dementia often lose their ability to communicate, pain management becomes very complex. Pain is a subjective sensation and not being able to communicate with persons with advanced dementia makes nurses uncertain of whether there is pain and, if so, whether it is being adequately managed. This corresponds to findings in the literature that pain assessment is challenging in people with dementia, because of their inability to understand and answer questions [29]. Even when nurses suppose that a person with dementia has pain, they find it difficult to assess the intensity and the kind of pain [30].

In this study, the nurses used either the Abbey Pain Scale assessment tool or no assessment tool at all. Many found the Abbey Pain Scale inadequate. These findings echo those of other studies where nurses in nursing homes have often found existing pain assessment tools inadequate [29, 31, 32]. This scepticism seems warranted. Lichtner and colleagues [18] assessed the reliability, validity and clinical utility of 28 pain assessment tools for people with dementia, including the Abbey Pain Scale, but could not recommend any particular pain assessment tool because of non-conclusive evidence. However, it is alarming that some nurses assess pain by intuition, without using assessment scales. The assessment and documentation of pain are needed in order to improve management of pain and should therefore be built into nursing homes care systems. Using an assessment scale instrument to evaluate pain routinely makes it more likely that the staff will notice pain and swiftly act accordingly. The National Board of Health & Welfare in Sweden also recommend use of scales such as Abbey Pain Scale and Doloplus 2 in this population, as pain is more likely to be recognised when routinely using an assessment scale, and because these scales are developed to assist assessment of pain in non-verbal individuals with end-stage dementia. They require minimal time to complete and are easy to understand. In addition, educational interventions for all nursing home staff, to improve knowledge and attitudes, could be a key and a significant step forward to increase the end of life care for frail older people in care homes but also for the possibility to evaluate interventions.

End-of-life care for residents with complex pain status and advanced dementia is a clinical and ethical challenge and the body of knowledge about pain management for this patient group is limited [13]. The administration of morphine injections becomes problematic for nurses when they are uncertain about the resident’s pain status. The most challenging aspect is finding the right balance between providing adequate pain relief and avoiding respiratory depression. Many nurses said that they would rather administer morphine with generosity than risk giving too little. This attitude is in line with a study of Brorson and colleagues [30], where nurses expressed a will to overtreat rather than undertreat pain, despite being mindful of the risk that they could cause a hastening of death. Residents with dementia in pain at nursing homes are often undertreated and require increasing dosages of opioids during the last week before death [13]. Therefore, opioid treatment (if possible, in oral form, otherwise transdermal patches or intravenous) should be initiated early at a low dose and titrated slowly along with registering adverse events [33]. Without doubt, pain is a personal experience influenced by many different factors, a combination of physical, emotional, social, and spiritual components, which has led to the concept of “total pain” [34].

A related issue involves the difficult clinical distinction between pain and anxiety. In line with previous findings [30, 35, 36], we found several nurses who experienced difficulty in distinguishing pain from anxiety. Likewise, Gilmore-Bykovskyi et al. [29] found that nurses feel uncertain about whether behavioural changes indicate pain or are related to other causes. Because the nurses were unable to define the exact cause of the behavioural change, administration of analgesia was delayed. Because of their difficulty in identifying pain, several nurses in our study combined midazolam injections for anxiety with morphine injections in the belief that midazolam also relieves pain. This drug combination used at the end of life has also been reported by Wilson and colleagues [37]. In another study, nurses stated that morphine alleviates both pain and anxiety [30]. Pain and anxiety appear to be associated and we believe further research should be carried out to better understand nurses’ decisions to use pain medication or psychotropic medication when treating pain [31].

Non-pharmacological interventions have received increasing attention in the palliative care literature in recent years; however, few nurses in this study mentioned such interventions. Because pain treatment remains inadequate for many residents with advanced dementia at the end of life, non-pharmacological interventions (such as music, aromatherapy, soft tissue massage) should be tried together with medication. We hypothesise that high workloads, lack of time among nurses or lack of knowledge are the reason for not prioritising non-pharmacological interventions, and as a consequence, not mentioning this topic in the interviews. Efforts to inform and educate may increase the use of these interventions.

### Relational challenges

The nurses in our study often mentioned relational challenges. To know and understand residents with advanced dementia, nurses must build good relationships with their relatives and be able to spend some time with the residents with advanced dementia [30, 36]. Lamaheva and colleagues [38] noted the importance of preparing early for a progressive decline in health and the inevitable end-of-life phase. Advance care planning can alleviate the burden of decision-making for both relatives and nurses who attempt to make the best decision for a resident with advanced dementia. Recurrently, the nurses in our study described relatives as a positive influence. Nevertheless, sometimes, mainly when nurses administered opioids at the end of life, the relatives’ opinions could be highly challenging. Then, nurses have to focus not only on the resident with advanced dementia but also on the well-being of their relatives. They often do so through conversations and by trying to be open to the relatives’ views and wishes [31]. We can see a need for some on-site educational support for relatives, as that could facilitate a better co-understanding of the situation for relatives and nurses. Because relatives should be included in palliative care programmes, as this is essential for understanding and learning about residents with advanced dementia, further research about cooperation between relatives and nurses should be promoted.

Pain treatment will be most effective if all components of pain are explored. When a person with advanced dementia cannot communicate verbally and are not able to exercise autonomy, relatives often need to act as proxies and advocates on their behalf. In our study, the nurses identified the relatives’ need for emotional support when trying to provide good care. If the relatives hold misconceptions, for example a belief that there is a risk of opioid addiction at the end of life, or a fear that the loved ones are going to die of opioids, it constitutes a barrier to good pain treatment. Persons with pain and advanced dementia at the end of life are therefore particularly vulnerable and very dependent on their caregivers. Nurses must therefore focus on reducing the distress and burdens for the person facing death, respecting the person’s dignity, and giving person-centred pain assessment and management, so that the correct dose can be found. Being free from pain is after all considered essential for good end-of-life care [39]. However, it is also within the nursing assignments to carefully explain, educate and have a discussion with the relatives about the symptoms and treatments accentuated at the end of life. For example, relatives need to be informed that an increase in morphine dosage at the end of life is generally related to the progression of disease, not to dependence developing. Likewise, when a person with advanced dementia has forced breathing, this can be a sign of pain. After opioids, respiration may slow down, for example from 24 breaths a minute to a stress-free 10 to 12 per minute, which is not a condition of life-threatening respiratory depression [40]. In cases were relatives ask for more generosity with opioids, nurses must act in line with the principle of non-maleficence and use opioids properly, not to harm the person in pain. A clear understanding of morphine treatment and its side effects is likely to promote confidence when administering morphine [37].

### Organisational challenges

The nurses also mentioned organisational challenges, especially in relation to good pain management. Because of economic barriers, insufficient nurse staffing has meant that an adequate nurse presence has not been achieved [41–43]. Slettebo and colleagues [44] found that although nurses wish to do more for people living with dementia and their relatives, insufficient nursing staff levels result in nurses prioritising physical needs such as pharmacological pain relief rather than psychosocial needs. This study indicates that insufficient staffing results in lonely residents with advanced dementia and could result in these people dying alone and in pain. This produced a feeling of powerlessness among the nurses. Previous studies have shown that people with dementia in a nursing home can suffer from the absence of relatives and caregivers at the end of life and can then, therefore, die in pain alone. This situation is ethically burdensome for nurses [45, 46] and should be taken seriously by care management and policymakers who set the frameworks for care.

Many residences with advanced dementia at nursing homes have complex needs and even though many dies in institutional care at nursing homes, they have not traditionally provided evidence-based palliative care, instead usually offering experience-based care [47]. Palliative care should be provided for people with life-threatening disease independent of diagnosis with a focus on early identification and assessment [21]. In recognising the importance of providing both high-quality general and specialised palliative care, the Swedish National Board of Health & Welfare has established evidence-based guidelines for palliative care in line with the WHO guidelines for palliative care [9]. These were later evaluated [48]. This evaluation found that municipalities responsible for the care of the elderly in some respects do not follow the National knowledge support for good palliative care. In order to empower nurses and nursing staff regarding management and assessment of  end-of-life pain, the authors suggest implementation of an end-of-life-care framework with support from external palliative care specialists. This is showed to improve the quality of end-of-life care for nursing home residents in UK [49]. Nevertheless, one challenge regarding support from external experts is to recognise when dying process in persons with dementia has started [50] and many health professionals reportedly tend to overestimate the remaining lifetime [51].

Specialist training in palliative care can also address the need for skills in ethical deliberation. Effective pain management in residents with advanced dementia at the end of life is often conceived as an ethical obligation and the strategies proposed are motivated by ethical reasoning. To give morphine with the intention of relieving distressing symptoms, even though there is a risk of thereby shortening the patient’s life, is often supported by “the doctrine of double effect”. This ethical principle says that “it is always wrong to do a bad act for the sake of good consequences, but it is sometimes permissible to do a good act knowing it might have some bad consequences” [52]. The application of this doctrine implies that doing something morally good (giving morphine to relieve pain) might potentially have a bad effect (shortening life), but that it is ethically permissible if the bad effect is not intended. In this way, healthcare professionals can use the principle to justifiably administer appropriate doses of morphine to a patient at the end of life, even though this could shorten the patient’s life. Used in this way, ethics training can provide conceptual tools that justify what intuitively feels right and help to distinguish justified from unjustified cases of high dosage administration.

To carry out individual expert assessments, we thus urgently need experienced, specialist nurses in nursing homes. The role of advanced nurse practitioner has been developed for a long time in the UK, US and Australia. These advanced nurse practitioners (also called advanced practice nurses or nurse consultants in the UK, clinical nurse specialists in the US, and clinical nurse consultants in Australia) are nurses with both theoretical and evidence-based knowledge who are expected to have expert skills in both complex decision-making and clinical judgement [53]. A position as an advanced nurse practitioner usually involves consultancy work as well as education and research work. While advanced clinical nursing is a common, well developed feature in many countries, Sweden has not facilitated the emergence of this profession. Based on the findings of this study, we suggest the implementation of advanced nurse practitioners for end-of-life dementia care. This would have the effect of improving patient outcomes, promoting continuity of care, supporting nursing and care teams, and stimulating research in the field [54].

### Strengths and limitations

The high workloads and lack of time among nurses affected our study, as we had hoped to interview several nurses from each nursing home. This was not, however, possible; only two informants were from the same nursing home, while the other 11 nurses all came from different workplaces.

Several limitations of this study should be taken into consideration. The aim from the beginning was to recruit a widely varied sample with respect to the nurses’ age, gender and work experience. However, because of poor recruitment and time pressure, all the nurses who volunteered and who met the inclusion criteria were included. A strategic sample would probably have provided a more varied and heterogeneous sample with participants who may have added data with wider variety (regarding age, gender and education level).

The participants were from nursing homes in one city, indicating that the results should not be extended to other countries and contexts. Nonetheless, we achieved saturation and good representation regarding age, gender and extent of work experience in Swedish nursing homes. However, it is noteworthy that none of the nurses had specialised in dementia, palliative or geriatric nursing.

Furthermore, there might be some risk of bias in that the head managers organised the recruitment of participants. It is possible that they opted for the most suitable participants, thereby masking potential problems.

Despite the limitations, we believe that this study contributes essential knowledge about the complexity of caring for residents with advanced dementia and pain at the end of life. There are still many gaps in our specific area, such as whether there are differences between nurses with long and short nursing experience and how pain management is affected in nursing homes that organise work differently. There is thus a need for further quantitative research, including further assessment of best practice for the end-of-life care of residents with advanced dementia and pain.

## Conclusions

This study shows that nurses face several demanding challenges when caring for persons with advanced dementia and pain at the end of life. One of the main issues was the difficulty in communicating with these persons, resulting in uncertain pain assessment. This results in difficulties in separating pain from anxiety and balancing the benefits and risks of morphine administration. Relatives can significantly influence the assessment and management of pain, both as interpreters of pain behaviour and by questioning the care given. Factors facilitating good palliative care and pain management included having good relationships with the other healthcare personnel, having extensive relevant professional experience, and having enough time to care for the resident with advanced dementia and their relatives.

The many challenges can affect the care of this growing and vulnerable group negatively and, therefore, it is crucial to promote more research in this area. We found that specifically trained specialist nurses are sorely needed at nursing homes in order to meet these challenges with the appropriate skills and knowledge. Additionally, there should be resources and strategies available for informing and involving family members in the care as they are often unfamiliar with the considerations involved in decisions (such as whether to administer morphine or not).

### Relevance to clinical practice

This study suggests that there is a need for trained nurses specialising in palliative care or dementia care at nursing homes in order to meet the challenges described with appropriate skills and knowledge. There is also a need for resources and strategies for informing relatives about end-of-life care and sometimes involving them in decision-making.

## Supplementary Information

**Additional file 1 **: **Table 2.** Interview guide.

## Data Availability

Additional data files in Swedish are available upon request to the corresponding author.

## Abbreviation

Nurses: Registered nurses
