# Supplementary material for: End-of-life care for people with advanced dementia and pain: a qualitative study in Swedish nursing homes
Source: BMC Nurs. 2021 Mar 20;20:48. doi: 10.1186/s12912-021-00566-7 (PMC7981921; doi:10.1186/s12912-021-00566-7)
Supplement: Supplementary file 1 — Additional file 1 : Table 2. Interview guide. [file 12912_2021_566_MOESM1_ESM.docx]

**Table 2. Interview guide**

| - Can you tell me how you assess pain in end-of-life care for residents with advanced dementia? - Can you tell me how you administer pain relief to residents with advanced dementia at the last phase of life? - Can you tell me a positive experience of successful pain management in a person with advanced dementia at the end of life? - Do you sometimes find it challenging to assess pain in residents with advanced dementia at the end of life? - Can you tell me about your experiences of providing pharmacological pain relief in residents with advanced dementia at the end of life? - What do you think the prerequisites are for giving good pain relief at the end of life? - Is there anything else you would like to tell me about pain assessment and pain relief in caring for residents with advanced dementia at the end of life? |
| --- |
